# The *Drosophila melanogaster* Na^+^/Ca^2+^ Exchanger CALX Controls the Ca^2+^ Level in Olfactory Sensory Neurons at Rest and After Odorant Receptor Activation

**DOI:** 10.3389/fncel.2018.00186

**Published:** 2018-07-03

**Authors:** Lorena Halty-deLeon, Bill S. Hansson, Dieter Wicher

**Affiliations:** Department of Evolutionary Neuroethology, Max Planck Institute for Chemical Ecology, Jena, Germany

**Keywords:** insect olfaction, *Drosophila melanogaster*, Na^+^/Ca^2+^ exchanger, CALX, Orco, calcium imaging

## Abstract

CALX, the Na^+^/Ca^2+^ exchanger in *Drosophila*, is highly expressed in the outer dendrites of olfactory sensory neurons (OSNs) which are equipped with the odorant receptors (ORs). Insect OR/Orco dimers are nonselective cation channels that pass also calcium which leads to elevated calcium levels after OR activation. CALX exhibits an anomalous regulation in comparison to its homolog in mammals sodium/calcium exchanger, NCX: it is inhibited by increasing intracellular calcium concentration [Ca^2+^]_i_. Thus, CALX mediates only Ca^2+^ efflux, not influx. The main goal of this study was to elucidate a possible role of this protein in the olfactory response. We first asked whether already described NCX inhibitors were capable of blocking CALX. By means of calcium imaging techniques in *ex-vivo* preparations and heterologous expression systems, we determined ORM-10962 as a potent CALX inhibitor. CALX inhibition did not affect the odor response but it affected the recovery of the calcium level after this response. In addition, CALX controls the calcium level of OSNs at rest.

## Introduction

Odorant receptor (ORs), a special class of olfactory receptors, have evolved in winged insects probably as a response to the challenge of detecting airborne odors (Missbach et al., 2014). These are composed by a specific ligand-binding subunit (OrX) and Orco, a highly conserved co-receptor protein (Larsson et al., 2004; Benton et al., 2006), which together form ligand-gated ion channels (Sato et al., 2008; Wicher et al., 2008). ORs are expressed in the dendrites of olfactory sensory neurons (OSNs) in the antennae and maxillary palps (Joseph and Carlson, 2015). Stimulation of ORs leads to ions fluxes across the OSN plasma membrane, including an influx of the ubiquitary second messenger Ca^2+^. Taking into account that the Ca^2+^ concentration outside the cells is around 10,000-fold higher than in the interior (Guerini et al., 2005) it is of vital importance to tightly regulate the free intracellular Ca^2+^ concentration [Ca^2+^]_i_. Thus, during and after agonist stimulation, the Ca^2+^ influx and efflux must be balanced in order to restore basal calcium levels.

There are two main Ca^2+^ extrusion systems working in parallel to maintain Ca^2+^ homeostasis (Guerini et al., 2005). These are the plasma membrane Ca^2+^-ATPase (PMCA) and the sodium/calcium exchanger (NCX). ATP-driven calcium pumps bind Ca^2+^ with high affinity but display a low turnover rate, NCX extrudes Ca^2+^ with a >10-fold higher turnover rate compared to PMCA pumps (Blaustein and Lederer, 1999). Consequently, at elevated intracellular calcium concentration ([Ca^2+^]_i_), e.g., during an action potential, the NCX is likely to be more efficient in extruding calcium than the PMCA.

In mammals, NCX acts as a bidirectional transporter. In its efflux (forward) mode, it exports one Ca^2+^ ion for the uptake of three Na^+^ first described in Nicoll et al. (1990). During pathological conditions or after agonist receptor stimulation and membrane depolarization, the reversed mode is triggered resulting in the uptake of Ca^2+^ and extrusion of Na^+^ (Blaustein and Lederer, 1999; Guerini et al., 2005; Lytton, 2007). Interestingly CALX, the NCX homolog in *Drosophila*, exhibits an anomalous regulation in that it is inhibited by increasing [Ca^2+^]_i_ (Hryshko et al., 1996). CALX shares 52% homology and a conserved structure with NCX. It is formed by 10 transmembrane helices with two intracellular calcium binding domains, CBD1 and CBD2 (Schwarz and Benzer, 1997). In contrast to NCX, CBD1 in CALX is the only calcium binding domain (Wu et al., 2010). Conformational changes as a result of Ca^2+^ binding to CBD1 are likely to be responsible for the Ca^2+^ inhibition of CALX (Wu et al., 2011). The physiological significance of this differential response to cytosolic Ca^2+^ remains to be elucidated. Since CALX is active only in its forward mode, little attention has been paid to its possible role in the sensory response even though it is the major Ca^2+^ extrusion system in *Drosophila* sensory neurons (Zheng et al., 2013). However, in *Drosophila* photoreceptor cells, previous studies have shown a role of CALX in amplification, adaptation and termination of the visual response (Wang et al., 2005; Wang and Montell, 2007). Its effect in other sensory modalities remains to be elucidated.

Due to the bimodal function of NCX, an inhibitor of the reversed mode could protect against Ca^2+^ overload in a situation of ischemia/reperfusion injury (Iwamoto, 2004). As a consequence, NCX inhibitors have attracted attention as potential Ca^2+^ regulators. The first NCX inhibitor described to block the reversed mode was KB-R973 (Iwamoto et al., 1996). Later on, SEA 0400 was developed as a more selective inhibitor by Matsuda et al. (2001). However, both compounds were seen to be not completely NCX-specific (Reuter et al., 2002). Recently, ORM-10962, a new selective inhibitor of the forward and reversed mode of NCX has been described (Kohajda et al., 2016).

Intracellular Ca^2+^ signaling modulates the signal amplification (Ignatious Raja et al., 2014) and the response profile (Fluegge et al., 2012) of the olfactory response. Bobkov et al. (2014) showed that the NCX inhibitor, KB-R7943, blocks odor-evoked activation in mosquito ORs expressed in heterologous expression system. Their results suggested that Orco could be a target for the drug action, raising the question of whether or not ORs could be linked directly or indirectly to a Na^+^/Ca^2+^ exchanger. Therefore, in the present study we asked whether CALX, as the major Ca^2+^ extrusion mechanism, could have a role in the odor response of OSNs in *Drosophila melanogaster*. We stimulated the OR expressing OSNs with the synthetic Orco agonist VUAA1 (Jones et al., 2011). In addition, it was crucial to use a selective CALX inhibitor. Using calcium imaging in *ex-vivo* preparations of fly antennae and a heterologous expression system, we tested three candidates for CALX inhibition: KB-R7943, SEA 0400 and ORM-10962. Among these, ORM-10962 was identified as a potent CALX inhibitor. Furthermore, we confirmed that CALX acts as the primary Ca^2+^ extrusion mechanism in *D. melanogaster* OSNs. Its major contribution to the odor response is restoring the basal calcium level after stimulation, with no significant further role in modulating the response.

## Materials and Methods

### Cell Culture and Transfection

*Drosophila melanogaster* Orco was cloned into pcDNA3.1(−) expression vector as previously described (Mukunda et al., 2014). HEK cells (DSMZ no. ACC 305) were purchased from the Leibniz Institute DSMZ GmbH (Braunschweig, Germany) and grown in DMEM/F12 1:1 medium (Gibco, Life Technologies, Grand Island, NY, USA) supplied with 10% Fetal Bovine Serum at 37°C and 5% CO_2_. HEK293 cells were electroporated with 1.6 μg Or83b-pcDNA3.1(−) using an Amaxa 4D-Nucleofector (Lonza GmbH, Cologne, Germany) with the SF Cell Line 4D-Nucleoefector X Kit (Lonza GmbH, Cologne, Germany). After electroporation, cells were cultured on poly-L-lysine (0.01%, Sigma-Aldrich, Steinheim, Germany) coated coverslips at a density of ~3 × 10^5^ cells per well (24 well plates). For experiments cells were exposed to normal bath solution (in mM: NaCl, 135; KCl, 5; MgCl_2_, 1; CaCl_2_, 1; HEPES, 10; d-glucose, 10; pH = 7.4; osmolarity 295 mOsmol/l).

### Fly Rearing and Antennal Preparation

*Drosophila melanogaster* flies with genotype *w;UAS-GCaMP6f/Cyo;Orco-Gal4/TM6B* were reared under a 12 h light: 12 h dark cycle at 25°C on conventional agar medium. For experiments, antennae of 4–8 days old females were excised and prepared as described in Mukunda et al. (2014). Briefly, flies were anesthetized on ice. Antennae were excised and fixed in vertical position with a two-component silicone and immersed in *Drosophila* Ringer solution (in mM: HEPES, 5; NaCl, 130; KCl, 5; MgCl_2_, 2; CaCl_2_, 2; and sucrose, 36; pH = 7.3) or Na^+^ free Ringer solution (in mM: HEPES, 5; N-Methyl-D-glucamine (NMDG), 130; HCl, 10; KCl, 5; MgCl_2_·6H_2_O, 2; Ca, 2; and sucrose, 30; pH = 7.3. Thereafter the funiculus was cut allowing access to the OSNs for experiments. Antennae were immersed in solution during the experiments.

### Calcium Imaging

Imaging was performed employing a monochromator (Polychrome V, Till Photonics, Munich, Germany), coupled to an epifluorescence microscope (Axioskop FS, Zeiss, Jena, Germany). A water immersion objective (LUMPFL 40× W/IR/0.8; Olympus, Hamburg, Germany) was used controlled by an imaging control unit (ICU, Till Photonics). Fluorescence images were acquired using a cooled CCD camera controlled by TILLVision 4.5 software (TILL Photonics). Experiments lasted 20 min with a sampling interval of 5 s. One-hundred microliter of the different chemicals were applied via pipette in proximity of the objective. VUAA1 was applied at a concentration of 25 μM, KB-R7943 at 100 μM, SEA 0400 at 0.1 μM and ORM-10962 at 1 μM. Samples were continuously perfused with bath solution in the perfusion/recording chamber (RC-27, Warner Instruments Inc., Hamden, CT, USA) during the experiments. TillVision software (Version 4.5, Till Photonics) was used to subtract background fluorescence and to define regions of interest (ROI) characterized by a change in the [Ca^2+^]_i_. Imaging experiments of cells were conducted 24 h after electroporation. Cells were incubated in Opti-MEM medium (Gibco) and loaded with 5 μM Fura-2 acetoxymethyl ester (Molecular Probes, Invitrogen) for 30 min at room temperature. After wash, cells were kept during the experiment in bath solution. Free intracellular Ca^2+^ concentration ([Ca^2+^]_i_) was calculated according to [Ca^2+^]_i_ = K_eff_(R − R_min_)/(R_max_ − R), where R_min_ and R_max_ were determined as described in Mukunda et al. (2014). Emitted light was separated by a 400 nm dichroic mirror and filtered with a 420 nm long-pass filter. Image pairs were obtained by excitation at 340 nm and 380 nm for 150 ms, background fluorescence was subtracted. The final resolution was 640 × 480 pixel in a frame of 175 μm × 130 μm. In antenna preparations, GCaMP6f was exited with 475 nm light at 0.2 Hz frequency with an exposition time of 50 ms. Emitted light was separated by a 490 nm dichroic mirror and filtered with a 515 nm long-pass filter. The response magnitude was calculated as the average ΔF/F_0_ in percentage following Mukunda et al. (2014).

### Chemicals

VUAA1 (N-(4-ethylphenyl)-2-((4-ethyl-5-(3-pyridinyl)-4H-1,2, 4-triazol-3-yl)thio)acetamide) was synthesized by the group “Mass Spectrometry/Proteomics” of the Max-Planck Institute for Chemical Ecology (Jena, Germany). KB-R7943 (2-[4-[(4-nitrophenyl)methoxy]phenyl]ethyl ester carbamimidothiotic acid, monomethanesulfonate) from Cayman Chemical (Ann Arbor, MI, USA). SEA-0400 (2-[4-[(2,5-difluorophenyl)methoxy]phenoxy]-5-ethoxyaniline) was purchased from ApexBio Tech LLC (Houston, TX, USA). ORM-10962 ([2-(4-hydroxy-piperidin-1-yl)-N-(6-((2-phenylchroman-6yl)oxy)pyridin-3-yl)acetamide]) was kindly provided by Orion Corporation (Orion Pharma, Espoo, Finland). All chemicals were dissolved in DMSO to yield a stock solution. When used as control DMSO was dissolved 1:1000.

### Immunolocalization

#### Antenna

Female flies between 4 days and 8 days old were collected. First, the head was removed and prefixed in 4% PFA (paraformaldehyde) +0.1% Triton for 10 min on ice. For preparation of the antenna, the 3rd segment was excised and fixed with 4% PFA (paraformaldehyde) +0.1% Triton for 2 h on ice. Antenna were washed with phosphate-buffered saline solution containing 0.1% Triton X-100 (PBST) for 3 × 20 min. Antennae were blocked in normal goat serum (NGS) in PBST (PBST-NGS) for 60 min at room temperature and then incubated with the primary antibodies in PBST-NGS for 2 days at 4°C. After 4 × 15 min wash at room temperature, antennae were incubated with secondary antibodies in PBST-NGS for 2 days at 4°C. Finally, antennae were washed again for 3 × 20 min and mounted in Vectashield (Vector, Burlingame, CA, USA). Primary antibodies used: mouse anti-GFP (A-11001, Invitrogen, Carlsbad, CA, USA), rabbit anti-CALX (provided by Dr. Craig Montell, University of California, USA). Secondary antibodies used: goat anti mouse Alexa 488- and goat anti rabbit Alexa 546 (A-11120, Invitrogen, Carlsbad, CA, USA).

#### Eyes

We prepared female flies (4 and 8 days old) according to Hsiao et al. (2012). Briefly, flies were anesthetized on ice, the head was removed and the retinas were dissected and fixed with 4% PFA (paraformaldehyde) for 15 min at room temperature. Afterwards, samples were washed with 1× phosphate-buffered saline solution (PBS) for 1 h. Retinas were blocked in 5% NGS in PBST for 20 min at room temperature followed by the incubation with the primary antibody overnight. After 3 × 15 min wash at with PBST, retinas were incubated with secondary antibodies in PBST-NGS overnight at room temperature. At last, retinas were washed with PBST for 1 h and mounted in Vectashield (Vector, Burlingame, CA, USA).

### Confocal Microscope

Images were acquired on a cLSM 880 (Carl Zeiss, Oberkochen, Germany) using a 40× water immersion objective (C-Apochromat, NA: 1.2, Carl Zeiss). Images were obtained at 0.10–0.10 μm intervals at 1592 × 1592 pixel resolution for the antennae overview and at 0.12–0.12 μm intervals at 512 × 512 pixel resolution for the detailed section. Confocal images were adjusted for contrast and brightness by using LSM Image Browser 4.0 (Carl Zeiss) and Adobe Photoshop CS.

### Data Analysis

Statistical analyses were performed using Prism 4 (Graph-Pad Software Inc., La Jolla, CA, USA). Data are given as mean ± SEM (standard error of the mean) and were analyzed using Paired *t*-test or Unpaired *t*-test. The decay was calculated and normalized in percentage during washing periods. Each washing period entails 150 s.

## Results

### Localization of CALX in OSN

**Figure 1:**
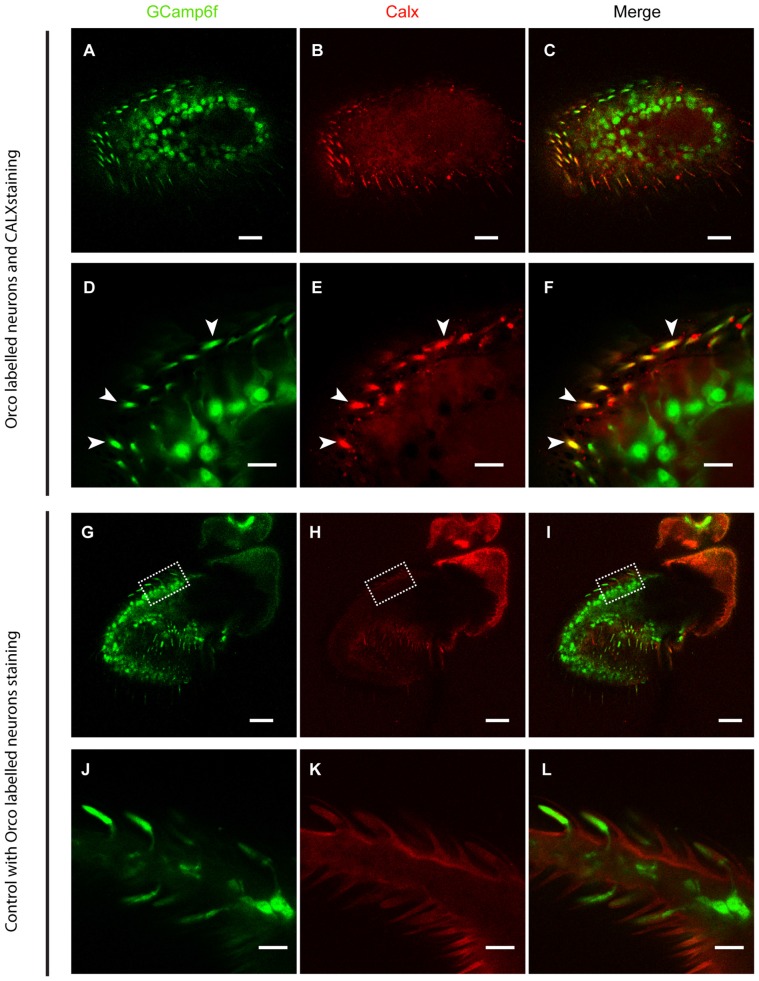
CALX is highly expressed in the outer dendrites of olfactory sensory neurons (OSNs). Superior panels: **(A,D)** GCaMP6f staining with Alexa 488. **(B,E)** CALX staining with Alexa 546. **(C,F)** Merged stainings. **(A–C)** General overview of female *Drosophila* antennae. Bars, 20 μm. **(D–F)** Details of a section at higher magnification, Bars, 5 μm. White arrows indicate localization of CALX in the dendrites. Lower panels: **(G,J)** GCaMP6f staining with Alexa 488. **(H,K)** Control staining with only Alexa 546. **(I,L)** Merged stainings. **(G–I)** General overview of female *Drosophila* antennae. Bars, 20 μm. **(J–L)** Details of a section at higher magnification, Bars, 5 μm.

As ORs are Ca^2+^-permeable, any receptor activation causes a Ca^2+^ influx. To ensure a fast and reliable Ca^2+^ handling after OR activation, we expected a high expression of CALX in the outer dendrites of OSNs, where ORs are expressed. To demonstrate this, we performed CALX immunostaining in the *Drosophila* antenna. Figure 1 shows that CALX indeed is mainly expressed in the outer dendrites whereas it is almost absent in the somata. To control for the specificity of anti-CALX we performed an immunostaining in the rhabdomeres of *Drosophila* eyes. The immunolocalization of CALX in photoreceptors cells had been previously shown by Wang et al. (2005), and our results are in agreement with their findings (for detail see Figure 1E in Wang et al., 2005 and Supplementary Figure S1 in this publication).

### KB-R7943 Attenuates Receptor Activation in *Drosophila melanogaster* OSNs

**Figure 2:**
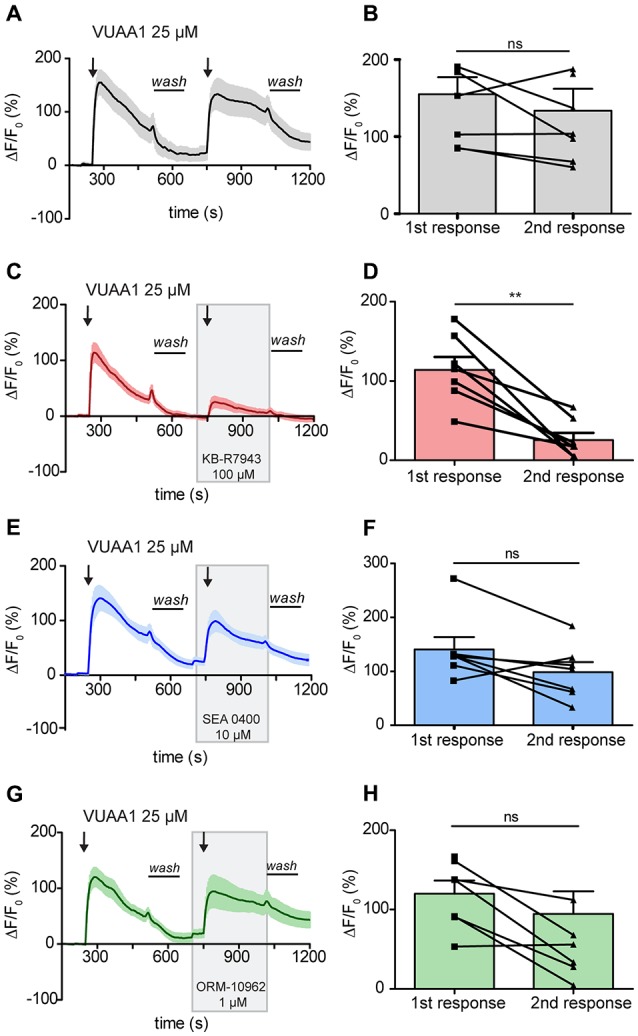
Activation of *Drosophila melanogaster* odorant receptors (ORs) is attenuated by KB-R7943. Left panels: averaged time course of the change in fluorescence intensity ΔF/F_0_ in *Drosophila* OSNs upon two stimulations with VUAA1 under control conditions (**A**, *n* = 8) and in the presence of KB-R7943 (**C**, *n* = 7), SEA 0400 (**E**, *n* = 7) and ORM-10962 (**G**, *n* = 7). Black arrows indicate application of 100 μl of VUAA1. Frames indicate the presence of sodium/calcium exchanger (NCX) inhibitors. *Wash* indicates the period of washing with Ringer solution between stimulations. Right panels **(B,D,F,H)**: maximum increase in ΔF/F_0_ after VUAA1 applications as in left panels. Data represent mean ± SEM, Paired *t*-test, ns, not significant, **p < 0.01.

In order to elucidate a possible role of CALX in the odor response, it was first necessary to determine a selective inhibitor that did not affect OR function. To determine if the previously described NCX inhibitors may act on ORs, we performed experiments using an *ex-vivo* preparation of *Drosophila* antennae. Two applications of the Orco agonist VUAA1 at 25 μM, one as control and one in the presence of the different compounds, were applied during the experiments. As a negative control, we performed the experiments only with applications of VUAA1. In control experiments there was no significant difference between the calcium fluorescence intensity of the first response (155 ± 22) compared to that of the second response (133.6 ± 28.7; Figures 2A,B; Paired *t*-test, ns > 0.05; *n* = 8). Our first candidate for CALX inhibition, KB-R7943, was already reported to block activation of ORs in the mosquito, and our results further supported this finding in *Drosophila* OSNs. The intensity of the response decreased from 114.2 ± 16.3 to 25.51 ± 9 after VUAA1 25 μM in the presence of KB-R7943 (100 μM; Figures 2C,D; Paired *t*-test, **p < 0.01; *n* = 7).

**Figure 3:**
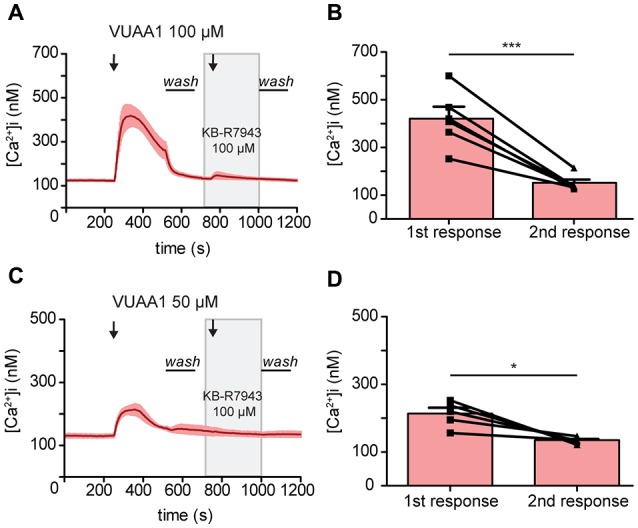
Activation of the odorant co-receptor Orco expressed in HEK293 cells is attenuated by KB-R7943. **(A,C)** Averaged time course of [Ca^2+^]_i_ upon stimulation with VUAA1 at different concentrations. (**A**, *n* = 6; **C**, *n* = 5). **(B,D)** Maximum increase in [Ca^2+^]_i_ as in **(A,C)**. Data represent mean ± SEM, Paired *t*-test, *p < 0.05, ***p < 0.001.

To confirm the blocking of Orco by KB-R7943, we then performed experiments in heterologous expression system. *Drosophila* Orco was expressed in Human Embryonic Kidney cells (HEK293) and the change in [Ca^2+^]_i_ was monitored with fura 2. The average increase in [Ca^2+^]_i_ decreased from 418.66 ± 47 to 146.67 ± 13.46 nM under VUAA1 100 μM conditions (Figures 3A,B; Paired *t*-test ***p < 0.001; *n* = 6). To exclude the possibility of Orco adaptation after a robust first response at VUAA1 100 μM, we repeated the experiments using a moderate stimulation of VUAA1 50 μM. Under this condition, the average increase in [Ca^2+^]_i_ also decreased significantly from 214.02 ± 16.94 to 135.03 ± 4.11 nM (Figures 3C,D; Paired *t*-test; *p < 0.05; *n* = 5). Hereby we confirmed that KB-R7943 is blocking Orco and therefore is not suitable to investigate a possible role of CALX in the odor response.

Our second candidate, SEA 0400, is a benzyloxyphenyl derivative, as KB-R7943, but was reported to be a much more potent NCX inhibitor (Matsuda et al., 2001). Due to the similarity to KB-R7943, we asked whether SEA 0400 could also block insect ORs. Wu et al. (2008) demonstrated that at a high concentration of 10 μM SEA 0400 was able to block the reverse mode of NCX in cultured rat embryonic cortical neurons. Therefore, we challenged *Drosophila* OSNs with SEA 0400 at 10 μM to test for a negative effect on Orco. In presence of SEA 0400 (10 μM), there was no significant difference between the intensity of the first response (140.6 ± 22.9) and the intensity of the second (98.34 ± 18.78; Figures 2E,F; Paired *t*-test, ns > 0.05; *n* = 7). Hence, SEA 0400 has no blocking effect on ORs and is a putative candidate for investigating the role of CALX in the odor response.

We then focused our attention in the last and most recently described NCX inhibitor: ORM-10962. This compound has been reported to be a new selective inhibitor of NCX in its reverse and forward mode at 1 μM (Kohajda et al., 2016). This fact made it particularly interesting since CALX is only functional in a forward mode. Our results show that this compound did not affect OR function. There was no significant difference in the OSNs response between control conditions (120 ± 16.6) and in the presence of ORM-10962 (94.62 ± 28.42; Figures 2G,H; Paired *t*-test; ns, *p* > 0.05; *n* = 7).

The lack of effect of SEA 0400 and ORM-10962 in the maximum of the odor response could indicate that: (i) these compounds do not act on CALX; or (ii) CALX does not affect the maximum of the response. However, as shown in Figures 2E,G, inhibition of CALX seems to affect the recovery of the Ca^2+^ level after washing off the OR ligand.

### CALX Shapes the Decay of the Ca^2+^ Level After an Odor Response

**Figure 4:**
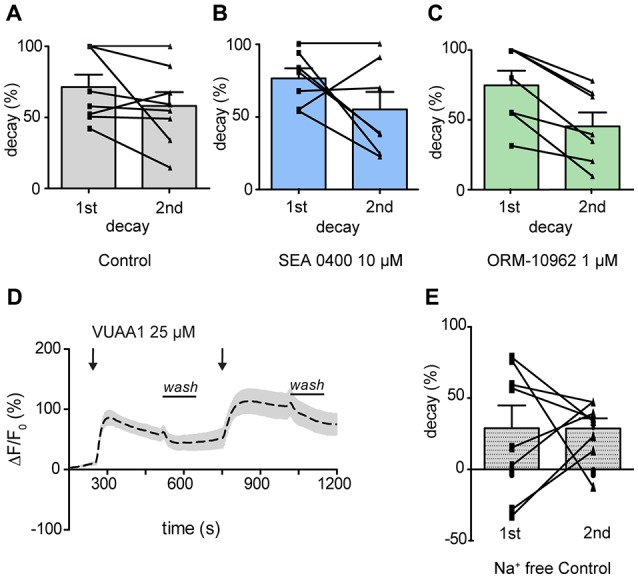
CALX function shapes the recovery of Ca^2+^ level after OR stimulation. Normalized decay in percentage during washing periods in the presence of VUAA1 under control conditions **(A)**, in presence of SEA0400 **(B)** and ORM-10962 **(C)**. In **(D)** averaged time course of the change in fluorescence intensity ΔF/F_0_ in *Drosophila* OSNs upon two stimulations with VUAA1 under Na^+^ free conditions (*n* = 8) and the correspondent normalized decay **(E)**. Data represent mean ± SEM.

We thus focused our attention on the decay of the response. To evaluate the contribution of CALX to Ca^2+^ level recovery, the decay of the response was calculated and normalized in percentage during washing periods (see Figure 2 for washing period). There was no significant difference between the decay after the first and the second response upon application of VUAA1 (25 μM) nor in the presence of SEA 0400 (10 μM; Figure 4A; Paired *t*-test; ns, *p* > 0.05; *n*_(VUAA1)_ = 8; Figure 4B; Paired *t*-test; ns, *p* > 0.05; *n*_(SEA)_ = 8). However, we observed a significantly lower decay, 45% compared to 74% under control conditions, after application of ORM-10962 (Figure 4C, Paired *t*-test; **p < 0.05; *n*_(ORM)_ = 7). To further test the influence of CALX in the decay of the Ca^2+^ levels, we performed the control experiments under Na^+^ free conditions (Figure 4D). Under the assumption that CALX function is impaired in the absence of Na^+^, we expected a reduced decay compared to the control experiment in normal conditions. This was indeed the case. The first (29%) and the second (29%) decay in Na^+^ free conditions (Figure 4E) are significantly lower compared to the first (71%) and the second (58%) decay under normal conditions (*t*-test 1st decay; *p < 0.05; *t*-test 2nd decay; *p < 0.05; *n*_(VUAA1)_ = 8, *n*(VUAA1_Na+ free_ = 8)). Furthermore, the decay in Na^+^ free conditions is comparable to the decay in presence of ORM (*t-test;* ns, *p* > 0.05; ns, not significant). These results point out that CALX plays a major role as a calcium extrusion mechanism and that it is involved in restoring Ca^2+^ levels after an odor response. It also provides a strong hint about the effectiveness of ORM-10962 as CALX inhibitor.

### ORM-10962 as CALX Inhibitor

**Figure 5:**
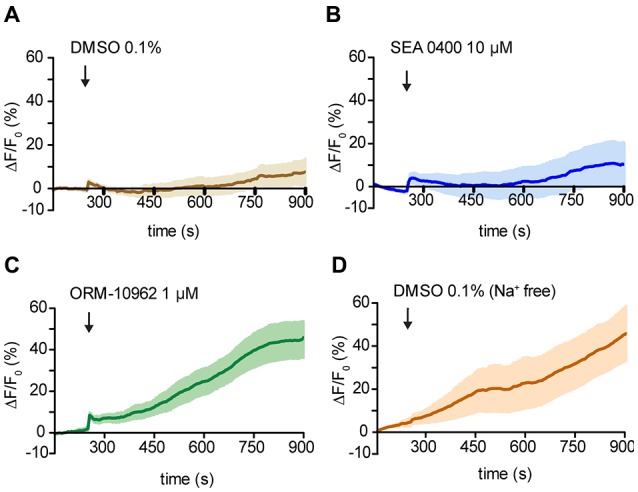
Effect of NCX inhibitors on resting Ca^2+^ fluorescence in OSNs. **(A—D)** Averaged fluorescence intensity over time in *Drosophila* OSNs after application of the chemicals (black arrows) under resting conditions. Control application of DMSO solution at 1% in *Drosophila* ringer **(A)** and in Na^+^ free ringer **(D)**. In **(B)** and **(C)** time course of the response to the NCX inhibitor SEA 0400 and ORM 10962 respectively. *n*_(DMSO)_ = 9, *n*(DMSO_Na+ free_) = 6, *n*_(SEA)_ = 9, *n*_(ORM)_ = 8.

To confirm ORM-10962 as a putative CALX inhibition, we performed experiments under resting conditions in the antenna preparation. In case of blocking the main Ca^2+^ extrusion mechanism, we would expect an increase in the [Ca^2+^]_i_ over time. For analysis, we compared the calcium levels at the end of the recording (900 s) between treatments and control (DMSO 0.1%; Figure 5A). In the presence of SEA 0400 there was no significant increase in the [Ca^2+^]_i_ compared to control conditions (*t*-test ns, *p* > 0.05; *n*_(DMSO)_ = 9, *n*_(SEA)_ = 9; Figure 5B). We hereby conclude that even though SEA 0400 has no negative effect on Orco channels it does not inhibit CALX. On the other hand, when ORM-10962 was applied, we observed a significant increase in [Ca^2+^]_i_ at 900 s (44.9 ± 8.8) compared to control conditions (7.8 ± 5.9; Figure 5C; *t*-test; **p < 0.01, *n*_(DMSO)_ = 9, *n*_(ORM)_ = 8). When we performed the control experiments under Na^+^ free conditions we observed an increase in the [Ca^2+^]_i_ over time (Figure 5D). This is in line with the assumption that under Na^+^ free conditions there is an impairment of CALX and therefore an accumulation of calcium is expected. Indeed, at 900 s we observed no significant difference between control in Na^+^ free (45.7 ± 13.3) and in the presence of ORM 1 μM (44.9 ± 8.8), *t*-test; *p* > 0.05; ns, not significant, *n*(DMSO_Na+ free_) = 7, *n*_(ORM)_ = 8). However, there is a significant difference between the quantified [Ca^2+^]_i_ between control and Na^+^ free conditions at 900 s (*t*-test with Welch’s correction, *p < 0.05, *n*(DMSO_Na+ free_) = 7, *n*_(DMSO)_ = 9).

Taken together, our results point out that ORM-10962 is capable of blocking the *Drosophila* Na^+^/Ca^2+^exchanger CALX without side effects on the co-receptor Orco and that CALX seems to play no role in the modulation of the odor response. However, according to our Na^+^ free and resting conditions experiments, CALX controls the Ca^2+^ level of OSNs at rest and is involved in shaping the recovery after odor stimulation.

## Discussion

Calcium entry following receptor activation in OSNs needs to be balanced to restore resting calcium levels in preparation for new stimuli. Calcium can be taken up by intracellular stores such as mitochondria and endoplasmic reticulum or extruded from the cell by Ca^2+^ pumps or exchangers. Sensory cascades operating through rapid Ca^2+^-mediated signaling seem to rely on Na^+^/Ca^2+^ exchange mechanisms. For example, *Drosophila* photoreceptor cells are very sensitive to perturbations in the Na^+^/Ca^2+^ exchange activity mediated by CALX (Wang et al., 2005). Furthermore, NCX was reported to be responsible for returning the concentration of intracellular Ca^2+^ to its basal level after odor stimulation in frog olfactory neurons (Reisert and Matthews, 1998). However, the involvement of CALX in the *Drosophila* odor response was so far unknown. The aim of the present study was to investigate this process.

Our immunohistochemistry results are in good agreement with previous studies where NCX was observed to be expressed in olfactory cilia (Noé et al., 1997; Danaceau and Lucero, 2000; Castillo et al., 2007) and dendrites (Jung et al., 1994). By measuring the change in calcium within the different neuronal compartments in *Xenopus*, Jung et al. (1994) observed an increase in calcium first in the dendritic compartments, whereas the increase in the soma and dendritic knob was delayed and less pronounced. Due to the fact that ATPase has a lower transport capacity for calcium than CALX, it seems plausible that CALX in the dendrites would be as a sink for calcium under conditions of elevated intracellular calcium concentration, such as after a receptor activation event, transporting calcium from the dendritic cytosol into the sensillum lymph.

In mammals, NCX is particularly important in cardiac myocytes. It has a key role in removing Ca^2+^ after excitation and contraction under normal conditions. However, it is also known to play an important role under pathological situations (Blaustein and Lederer, 1999; Iwamoto et al., 2004). In the case of arrhythmias, the reversed mode of NCX could lead to a Ca^2+^ overload (Sipido et al., 2007). The development of NCX inhibitors has therefore been targeted as a strategy to study regulatory calcium mechanisms. In contrast to KB-R7943 and SEA 0400, where both compounds preferentially block the reverse mode of NCX (Iwamoto et al., 2004), ORM-10962 acts on the two opposite NCX operational modes (Kohajda et al., 2016). Yet, no inhibitor of the *Drosophila* Na^+^/Ca^2+^ exchanger CALX had been described. To understand a possible role of CALX in *Drosophila* olfactory transduction, it was crucial to selectively block it independently of other elements in the transduction cascade. Given evidence of three NCX inhibitors, we studied these compounds as potential blockers of CALX.

The first two compounds, KB-R7943 and SEA 0400, are amiloride derivatives. Besides mainly blocking the reverse mode of NCX, amiloride derivatives have been shown to block odorant-evoked activity in lobster olfactory receptor neurons (Bobkov and Ache, 2006). Specifically, KB-R7943 blocked the olfactory response in lobster (Pezier et al., 2009) and mosquito (Bobkov et al., 2014). In both studies, inhibition of the olfactory response was almost total between 50 μM and 100 μM of KB-R7943. Accordingly, our experiments confirmed that KB-R7943 attenuated the activation of *Drosophila* ORs significantly (Figure 2). In addition, our results in HEK cells strongly suggest that KB-R7943 acts on the co-receptor Orco directly (Figure 3). This is further supported by the fact that—when testing the other putative inhibitors, namely SEA 0400 or ORM-10962—we observed no attenuation in the Orco response (Figure 2). Our data indicate that KB-R7943 blocks the co-receptor Orco, and hence cannot be used to study the role of CALX in olfaction.

In contrast to KB-R7943, SEA 0400 appear to have no side effect on Orco (Figures 2E,F). The minor, insignificant inhibition could be due to a weak specificity for NCX as stated by Reuter et al. (2002). This result, together with the fact that Bouchard et al. (2004) postulated a state-dependent inhibition of NCX by SEA 0400, made this compound a putative CALX inhibitor. However, although SEA had been reported to be more selective for NCX (Matsuda et al., 2001) and being 30 times more potent than KB-R7943 (Iwamoto et al., 2004), our results indicate that it is not potently acting on CALX. Even at a high concentration of 10 μM SEA 0400 failed to inhibit the forward mode of the exchanger (Figure 5B). Iwamoto et al. (2004) showed that SEA 0400 preferentially inhibits the reverse mode of mainly NCX1 but not the other NCX isoforms (NCX2 and NCX3) at concentrations between 10 nM and 1 μM. Such isoform specificity could be the reason for the lack of effect on CALX. Nonetheless, the calcium binding domain (CBD1) in CALX and NCX share 60% sequence identity (Wu et al., 2010). Therefore, the lack of effect on CALX could be attributable to the absence of a reverse mode in CALX or the reduced specificity mentioned before.

The experiments with the last candidate for inhibition of CALX, ORM-10962, indicated that there was no negative effect in the Orco-response (Figures 2G,H). By contrast, the decay of the Ca^2+^ signal back to baseline was significantly altered (Figure 4C). This indicates that the restitution of the Ca^2+^ levels in the presence of ORM-10962 was impaired, which is confirmed by our experiments in resting conditions (Figure 5C). The importance of CALX in restoring calcium levels is also highlighted by our results under Na^+^ free conditions (Figures 4D,E). Under this circumstance, CALX function is impaired and thus the decay of the first and the second response is comparable to the decay in presence of ORM-10926. Elevated calcium levels could be reduced by efflux through the plasma membrane by Na^+^/Ca^2+^ exchange and/or the PMCAs. Previous studies reported that NCX acts as the major Ca^2+^ extrusion mechanism in frogs (Jung et al., 1994; Reisert and Matthews, 1998) and mouse (Noé et al., 1997) olfactory response. However, Castillo et al. (2007) suggested that PMCA could also play an important role in restoring calcium basal levels in rat (Sprague–Dawley) and toad (*Caudiverbera caudiverbera*) olfactory neurons. They argued that because of its lower affinity to calcium and its voltage dependent properties, NCX’s efficiency will decline with depolarization of the neurons during an odor response. Their evidence suggests that both Ca^2+^ transporters contribute to re-establish resting Ca^2+^ levels in the cilia following olfactory responses. However, our results suggest that in *Drosophila*, CALX plays a more important role in maintaining calcium homeostasis (Figure 5). Calmodulin, a Ca^2+^ binding protein, modulates *Drosophila* odorant receptor function through Orco (Mukunda et al., 2014) and is able to potentiate the action of PMCA in olfactory cilia (Castillo et al., 2007). Hence, the slower decay observed in the presence of ORM after stimulation of Orco could be due to the action of PMCA (Figure 4). Further experiments to investigate these processes will be important to shed more light into Ca^2+^ regulatory mechanisms in *Drosophila* olfactory transduction.

In conclusion, in the current study we identified ORM-10962 as potent CALX inhibitor. As in other organisms, where Na^+^/Ca^2+^ exchangers are important for the dynamics of the olfactory response (Jung et al., 1994; Noé et al., 1997; Reisert and Matthews, 1998; Danaceau and Lucero, 2000; Castillo et al., 2007), CALX appears to function as the major calcium extrusion mechanisms in *Drosophila* olfactory neurons both under resting conditions and after enhanced activity.

## Ethics Statement

This study on the vinegar fly *Drosophila melanogaster* was performed in Germany where the research on invertebrates does not require a permit from a committee that approves animal research.

## Author Contributions

DW and LH-L designed the experiments. LH-L conducted the experiments and the analysis. LH-L, DW and BH wrote the article.

## Conflict of Interest Statement

The authors declare that the research was conducted in the absence of any commercial or financial relationships that could be construed as a potential conflict of interest.

## Supplementary Material

FIGURE S1 CALX staining in the eye of *Drosophila melanogaster*. Staining of CALX in the rhabdomeres with Alexa 546. Bar, 5 μm.
