# Supplementary figures and images for: The Drosophila melanogaster Na+/Ca2+ Exchanger CALX Controls the Ca2+ Level in Olfactory Sensory Neurons at Rest and After Odorant Receptor Activation
Source: Front Cell Neurosci. 2018 Jul 3;12:186. doi: 10.3389/fncel.2018.00186 (PMC6038709; doi:10.3389/fncel.2018.00186)

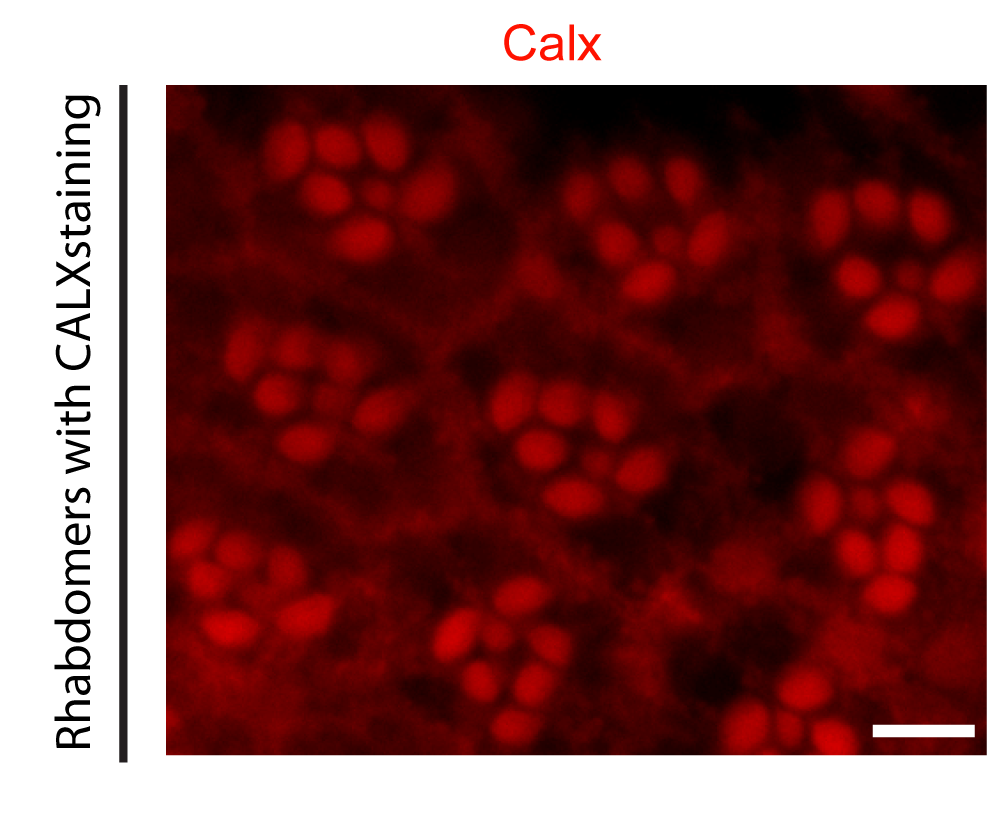

Supplement: FIGURE S1 — CALX staining in the eye of Drosophila melanogaster. Staining of CALX in the rhabdomeres with Alexa 546. Bar, 5 μm. [file Image_1.TIF]
